# The Impact of Psychiatric conditions on Functional Gastrointestinal Symptoms

**DOI:** 10.1192/j.eurpsy.2025.1019

**Published:** 2025-08-26

**Authors:** K. Imen, R. Nakhli, M. Bouhoula, L. S. Chaibi, O. Ajmi, N. Belhadj, A. Aloui, M. Kahloul

**Affiliations:** 1 Occupational Medicine Department, University of Sousse, Faculty of Medicine Ibn Al Jazzar; 2 Occupational Medicine Department (LR19SP03), Faculty of Medicine of Sousse, Farhat Hached University Hospital, University of Sousse, Sousse; 3 D Psychiatric Department, Razi Hospital, Tunis; 4 Psychiatry Department, Psychiatry Department, Farhat Hached University Hospital Medicine Department, Sahloul University Hospital; 5 Occupational Medicine Department, Occupational Medicine Department, Sahloul University Hospital; 6 4. Anesthesia and Intensive Care Department, Sahloul University Hospital, Sousse, Tunisia

## Abstract

**Introduction:**

Functional gastrointestinal disorders (FGIDs) represent a significant global health burden, affecting approximately 15-20% of the population. These disorders can significantly impair quality of life, particularly in individuals facing high levels of psychological stress, such as medical students.

**Objectives:**

To study the association between psychiatric factors, specifically anxiety and depressive disorders, and the prevalence of FGIDs among medical students in Tunisia.

**Methods:**

A cross-sectional study was conducted among second-year medical students at the Faculty of Medicine, Sousse, from March 2023 to February 2024. Data were collected using a self-administered questionnaire designed to assess gastrointestinal symptoms such as abdominal pain, bloating, diarrhea, constipation, and heartburn. Psychiatric conditions were assed using validated screening tools.

**Results:**

The study included 206 students, with a strong female predominance (80.1%). Among them, 46.1% (n=95) reported experiencing between 1 and 4 digestive disorders, primarily abdominal pain and bloating (66%). Psychiatrically, univariate analysis revealed a significant association between FGIDs and several psychological factors: female gender (p<0.01), a tendency towards anger in daily life (p<0.01), anticipatory anxiety (p<0.01), and the presence of panic attacks (p<0.01).

**Conclusions:**

Functional gastrointestinal disorders (FGIDs) are prevalent among medical students and are often linked to anxiety and depressive disorders. Preventive measures focused on stress management and mental health could significantly improve the quality of life of these students and prevent digestive complications.

**Disclosure of Interest:**

None Declared

